# ANGPTL3 Inhibition With Evinacumab Results in Faster Clearance of IDL and LDL apoB in Patients With Homozygous Familial Hypercholesterolemia—Brief Report

**DOI:** 10.1161/ATVBAHA.120.315204

**Published:** 2021-03-11

**Authors:** Laurens F. Reeskamp, John S. Millar, Liya Wu, Hans Jansen, Dewi van Harskamp, Henk Schierbeek, Daniel A. Gipe, Daniel J. Rader, Geesje M. Dallinga-Thie, G. Kees Hovingh, Marina Cuchel

**Affiliations:** 1 Department of Vascular Medicine (L.F.R., G.K.H.), Amsterdam UMC, location AMC, University of Amsterdam, The Netherlands.; 2 Department of Experimental Vascular Medicine (H.J.), Amsterdam UMC, location AMC, University of Amsterdam, The Netherlands.; 3 Stable Isotope Research Laboratory, Endocrinology, Vrije Universiteit (D.v.H., H.S.), Amsterdam UMC, location AMC, University of Amsterdam, The Netherlands.; 4 Institute for Diabetes, Obesity, and Metabolism (J.S.M.), Perelman School of Medicine, University of Pennsylvania, Philadelphia.; 5 Division of Translational Medicine and Human Genetics, Department of Medicine (J.S.M., L.W., D.J.R., M.C.), Perelman School of Medicine, University of Pennsylvania, Philadelphia.; 6 Regeneron Pharmaceuticals, Inc, Tarrytown, NY (D.A.G.).

**Keywords:** angiopoietin-like protein, cholesterol, LDL, familial hypercholesterolemia, isotope, leucine, lipoprotein

## Abstract

**Objective:**

The mechanism by which evinacumab, a fully human monoclonal antibody directed against ANGPTL3 (angiopoietin-like 3 protein) lowers plasma LDL (low-density lipoprotein) cholesterol levels in patients with homozygous familial hypercholesterolemia is unknown. We investigated apoB (apolipoprotein B) containing lipoprotein kinetic parameters in patients with homozygous familial hypercholesterolemia, before and after treatment with evinacumab.

**Approach and Results:**

Four patients with homozygous familial hypercholesterolemia underwent apoB kinetic analyses in 2 centers as part of a substudy of a trial evaluating the efficacy and safety of evinacumab in patients with homozygous familial hypercholesterolemia. The enrichment of apoB with the stable isotope (5,5,5-^2^H_3_)-Leucine was measured in VLDL (very LDL), IDL (intermediate-density lipoprotein), and LDL at different time points before and after intravenous administration of 15 mg/kg evinacumab. Evinacumab lowered LDL-cholesterol by 59±2% and increased IDL apoB and LDL apoB fractional catabolic rate in all 4 homozygous familial hypercholesterolemia subjects, by 616±504% and 113±14%, respectively. VLDL-apoB production rate decreased in 2 of the 4 subjects.

**Conclusions:**

In this small study, ANGPTL3 inhibition with evinacumab is associated with an increase in the fractional catabolic rate of IDL apoB and LDL apoB, suggesting that evinacumab lowers LDL-cholesterol predominantly by increasing apoB-containing lipoprotein clearance from the circulation. Additional studies are needed to unravel which factors are determinants in this biological pathway.

**Registration:**

URL: https://www.clinicaltrials.gov; Unique identifier: NCT04722068.

## Abstract 2

Supplemental Digital Content is available in the text.

**Highlights**

- ANGPTL3 (angiopoietin-like 3) inhibition with evinacumab is very effective in reducing LDL (low-density lipoprotein) cholesterol in patients with homozygous familial hypercholesterolemia.
- ANGPTL3 inhibition with evinacumab markedly increases the fractional catabolic rate of IDL (intermediate-density lipoprotein)- and LDL-apoB in 4 patients with homozygous familial hypercholesterolemia.
- These results suggest that evinacumab lowers LDL cholesterol predominantly by increasing apoB-containing lipoprotein clearance from the circulation.

ANGPTL3 (angiopoietin-like 3 protein) is a regulator of lipoprotein metabolism and has recently emerged as a novel therapeutic target to treat patients with dyslipidemia. Carriers of loss-of-function (LOF) variants in *ANGTPL3* present with low triglyceride, low LDL (low-density lipoprotein) cholesterol (LDL-C), and low HDL (high-density lipoprotein) cholesterol (HDL-C) plasma levels,^1^ as well as a decreased risk for cardiovascular disease compared with non-carriers.^2,3^

ANGPTL3 is mainly expressed in the liver and has been shown to reduce the activity of LPL (lipoprotein lipase)^4^ and EL (endothelial lipase) in vitro,^5^ which is widely considered to explain the observed low triglyceride and HDL-C levels, respectively, in carriers of LOF variants in *ANGPTL3.* Hitherto, it is not fully elucidated which mechanism underlies the effect of ANGPTL3 on LDL cholesterol metabolism. Animal models and in vitro studies have shown that ANGPTL3 has an effect both on production as well as clearance of apoB (apolipoprotein B) containing lipoproteins.^6,7^ Interestingly, the LDL-C lowering effects of ANGPTL3 inhibition with either monoclonal antibodies or antisense oligonucleotides seems to be LDL receptor (LDLR) independent since marked LDL-C lowering is observed in both *Ldlr* knock-out (KO) mice^6,8^ and patients with homozygous familial hypercholesterolemia (hoFH).^9^ Recent reports of studies in mice suggest that EL is required for ANGPTL3 inhibition to reduce LDL-C levels in absence of a functional LDLR.^10,11^

To further elucidate the physiological effect of ANGPTL3 inhibition on LDL-C lowering, we investigated apoB-containing lipoprotein kinetics in 4 patients with hoFH before and after treatment with evinacumab, a fully human monoclonal antibody against ANGPTL3.

## Materials and Methods

The data that support the findings of this study are available from the corresponding author upon reasonable request.

### Study Population and Clinical Protocol

We invited patients with hoFH who were enrolled in an open-label, single-arm study assessing the efficacy and safety of evinacumab in patients with hoFH^9^ to also participate in a substudy to evaluate the production and catabolic rates of apoB-containing lipoproteins before and after receiving evinacumab. In brief, patients ≥18 years old with genetically confirmed hoFH were eligible for inclusion if they had LDL-C levels above 70 mg/dL while on stable lipid-lowering therapy for at least 4 weeks for statins and ezetimibe, 8 weeks for PCSK9 inhibition, and 12 weeks for lomitapide. Four subjects (2 at Amsterdam UMC [AUMC] and 2 at University of Pennsylvania [UPENN]) underwent apoB kinetic measurements before the first dose of study drug was given (baseline) and 1 week (subject AUMC_1) or 6 weeks (all other subjects) after receiving one IV dose of evinacumab (treatment). Evinacumab was administered intravenously at a dose of 15 mg/kg. All subjects gave informed consent for participation in the substudy and the parent study. All studies were approved by the medical ethic committees of the 2 research institutes (Amsterdam UMC and University of Pennsylvania).

### Study Protocol

Subjects fasted overnight (>10 hours) before the study. (5,5,5-^2^H_3_)-Leucine was administered via a venous catheter as 7 mg/kg bolus (AUMC) or as primed (1.34 mg/kg) continuous 12-hour infusion (1.34 mg/kg per hour; UPENN). Blood samples were drawn at multiple timepoints for 24 to 48 hours for the determination of (5,5,5-^2^H_3_)-leucine enrichment of apoB in VLDL (very low-density lipoprotein), IDL (intermediate-density lipoprotein), and LDL fractions. The 2 subjects who were enrolled at the AUMC received a standardized meal (whole wheat bread with light cheese) 2 hours after bolus infusion and dinner ad libitum in the evening. After a 10 hours admission, patients went home and the blood sample at t=24 hours was collected at the patients’ home by a trained trial nurse. The 2 subjects enrolled at UPENN remained in the research unit overnight and were maintained in a constant fed condition for the first 20 hours of the study by receiving their total daily caloric intake in the form of 10 identical small meals every other hour starting 1 hour prior the start of the infusion. They were discharged after the 24-hour time point and returned at the research unit for a 48-hour blood draw.

### Laboratory Methods

We measured the incorporation of (5,5,5-^2^H_3_)-leucine in the apoB moiety in VLDL, IDL, and LDL. At the AUMC site, VLDL, IDL, and LDL fractions were isolated from plasma by a 1-step gradient ultracentrifugation using a SW41 rotor (Beckman). In short, the density of 3.5 mL of plasma was adjusted to 1.25 g/mL with 2.695 g KBr. A total of 3.0 mL plasma (d=1.25 g/mL) was transferred to an ultra-clear Beckman SW41 tube. The gradient was formed by layering the following salt solutions on top of the plasma: (1) 2 mL d=1.225 g/mL; (2) 4 mL d=1.100 g/mL; (3) 3 mL d=1.006 g/mL. The different fractions were then isolated by centrifugation in a Beckman ultracentrifuge 29 000 rpm, 10 °C, 19 hour and termination without brake. Fractions were frozen and stored at −80 °C for further analysis. For leucine enrichment analysis of apoB VLDL, IDL and LDL fractions were precipitated with isopropanol, delipidated with ethanol-diethyl ether, dried, and hydrolyzed with 6 mol/L HCl at 110 °C for 24 hours.^12^ The samples were then prepared for analysis of leucine enrichment as described using norleucine as internal standard. Enrichments were determined by GC-MS GC-MSD5975c (Agilent Technologies, Amstelveen, The Netherlands) equipped with a VF17 ms column operated in SIM mode. For the correction and calculation of obtained isotope enrichments, the average values of the m/z 161:158 ratio were determined using a calibration curve with known quantities of labeled and unlabeled leucine.^13^ The resulting m/z 161:158 was expressed as molar percentage ratio.^13^

At the UPENN site, the enrichment of VLDL, IDL, and LDL apoB was determined as previously described.^14^ Briefly, lipoprotein fractions were isolated from plasma by sequential ultracentrifugation. ApoB100 was isolated from VLDL, IDL, and LDL by SDS-PAGE. ApoB100 bands were hydrolyzed using 6 N HCl followed by derivatization of amino acids to their heptafluorobutyryl isobutyl esters. Leucine isotope enrichments were determined in the IDOM Metabolic Tracer Resource at UPENN using GC-MS.^15^

### ApoB Kinetic Modelling and Parameter Estimation

Fractional transfer and catabolic rates for apoB were determined by fitting the tracer data to a previously described multi-compartmental model^15^ (Figure I in the Data Supplement) using the WinSAAM modeling program. The precursor plasma D3-leucine enrichment data were modeled as a forcing function for newly secreted apoB. Clearance from the VLDL and IDL remnants and LDL pools were fit using Bayesian estimation to improve parameter identifiability. On-treatment values for these parameters were set to the same values obtained during the baseline treatment period and were multiplied by another parameter that allowed them to increase, if necessary. VLDL, IDL, and LDL apoB concentrations were either measured directly (patients UPENN_1, UPENN_2) or calculated as a percentage of the total plasma apoB concentration (patients AUMC_1, AUMC_2). Pool sizes were determined by multiplying the apoB concentration in each fraction (mg/dL) by the estimated plasma volume (body weight in kg×0.45 dL/kg). Production rates were calculated by multiplying fractional transfer and catabolic rates by the corresponding pool size and expressed relative to body weight.

### Statistical Methods

This is a descriptive study with a small sample size. Therefore, no formal statistical testing was performed. Results obtained from each subject are reported individually and summarized as mean±SD, if normally distributed, or as median (inter quartile range), if not normally distributed. All analyses were performed in R version 3.6.1 (The R Foundation, Vienna, Austria).

## Results

### Subject Characteristics

The characteristics of the 4 adult patients with hoFH are depicted in Table 1. The 2 subjects enrolled at the AUMC were compound heterozygous for *LDLR* defective variants (Table) and presented with less severe hypercholesterolemic phenotypes compared with the 2 UPENN subjects who were shown to carry 2 *null* variants in *LDLR* (Table 1). Both UPENN subjects stopped lipoprotein apheresis at least 4 weeks before the baseline study. Background lipid-lowering therapy consisted of a statin, ezetimibe, and a PCSK9 inhibitor in 3 subjects and of lomitapide in the fourth subject (UPENN_2) and had been stable as required per protocol, in all participants.

**Table 1.**
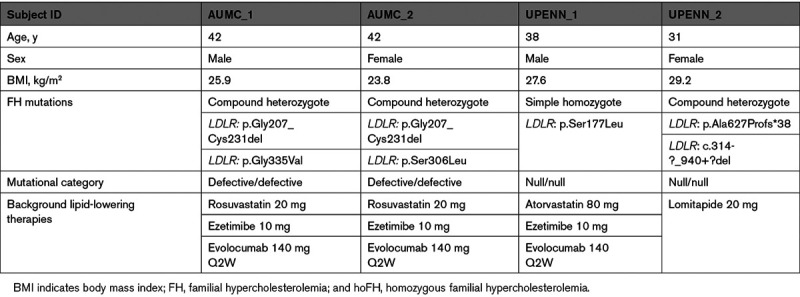
Characteristics of Patients With hoFH at Baseline

**Table 2.**
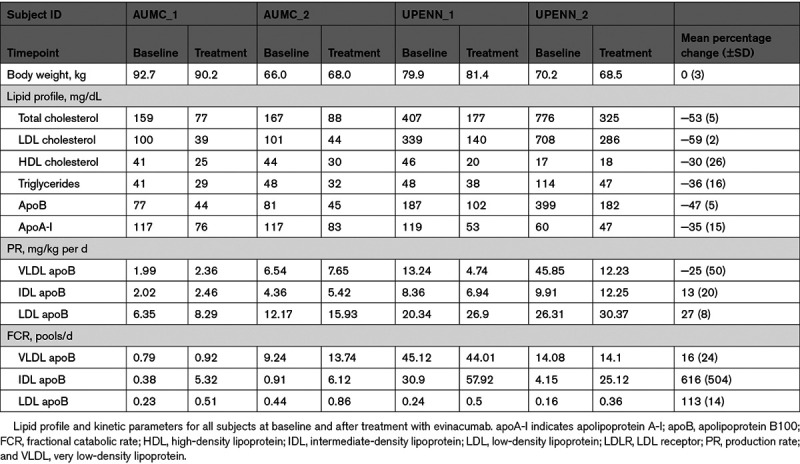
Lipid Profile and Kinetic Parameters for All Subjects at Baseline and After Treatment With Evinacumab

### Treatment Effect of Evinacumab on Lipid Profile

Exposure to one infusion with the ANGPTL3 antibody evinacumab (15 mg/kg) resulted in pronounced decreases (mean percent change ±SD) in plasma levels of total cholesterol (−53±5%), LDL-C (−59±2%), HDL-C (−30±26%), triglycerides (−36±16%), apoB (−47±5%), and apoA-I (−35±15%; Table 2). No serious adverse events occurred during this kinetic substudy.

### Treatment Effect on apoB Production and Catabolic Rates

To estimate the effects of evinacumab treatment on apoB turnover, we analyzed the rates at which the stable isotope leucine was incorporated into and removed from apoB in the VLDL, IDL, and LDL fractions. Upon evinacumab treatment apoB concentrations decreased in VLDL, IDL, and LDL by 41±38%, 81±11%, and 40±7%, respectively (Table I in the Data Supplement). The effect of evinacumab on the production rate of apoB in the VLDL, IDL, and LDL fractions was variable among the participants (see Figure and Table 2, Figures II and IIIA through IIID in the Data Supplement for individual results) and showed mean percent changes of −25±50%, 13±20%, and 27±8%, for VLDL, IDL, and LDL, respectively. The effect on fractional catabolic rate (FCR) was more pronounced and consistent. ApoB FCR increased by 16±24%, 616±504%, and 113±14% in VLDL, IDL, and LDL fractions, respectively (see Figure and Table 2, Figures II and IIIA through IIID in the Data Supplement for individual results).

**Figure.**
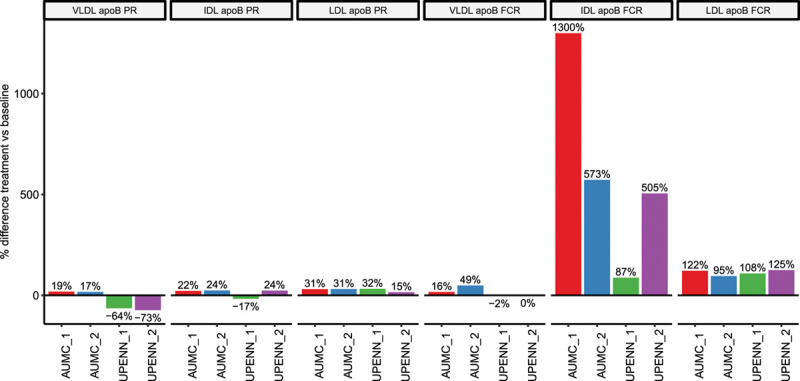
**Percentage change between treatment and baseline apoB (apolipoprotein B100) production and fractional catabolic rates for lipoprotein subfractions.** FCR indicates fractional catabolic rate; IDL, intermediate-density lipoprotein; LDL, low-density lipoprotein; PR, production rate; and VLDL, very-low density lipoprotein.

## Discussion

This is the first study to investigate the effects of ANGPTL3 inhibition with a fully human monoclonal antibody on apoB kinetics in humans. In the 4 patients with hoFH evaluated, inhibition of ANGPTL3 by evinacumab resulted in marked increases in FCRs of IDL and LDL apoB. The effect on the apoB production rates of the lipoprotein subfractions was less clear, with 2 of the 4 participants showing a decrease in the production rate of VLDL apoB, and a small increase of the production rate of IDL- and LDL apoB noted in all 4 subjects. Although we acknowledge the very small number of subjects studied, the heterogeneity of their phenotypic and genotypic characteristics, and the difference in the kinetic study protocol, these data suggest that the decrease in LDL-C plasma concentrations observed following evinacumab administration is mainly due to an increased catabolism of the IDL and LDL fractions.

Our results are in line with an earlier published apoB kinetic study in a family with familial hypobetalipoproteinemia,^16^ which was later reclassified as familial combined hypolipidemia caused by *ANGPTL3* LOF variants.^1^ The affected family members presented with lower VLDL apoB production rates, and increased IDL and LDL apoB FCRs compared with unaffected family controls,^16^ with a clear gene dose effect.^1^

Possible mechanisms underlying the effect of ANGPTL3 inhibition on LDL-C could be related to an enhanced clearance and/or reduced production of LDL precursors. Given the known inhibitory effects of ANGPTL3 on LPL,^4^ it is possible that the reduced levels of LDL-C are at least in part due to increased lipolytic activity of LPL and consequent accelerated clearance of apoB containing particles from the circulation via LPL and non-LPL-mediated pathways. We observed a substantial increase in IDL apoB FCR in all 4 subjects with only a slight increase in LDL apoB production rate, indicating that IDL is mostly cleared from the circulation. This is consistent with the minimal effects in LDL apoB production rate in the family carrying *ANGPTL3* LOF.^1^ The known effect of ANGPTL3 on LPL cannot, however, by itself explain the observed increase in IDL and LDL apoB FCR.

ANGPTL3 is also known to inhibit EL.^5^ EL is well known to affect HDL metabolism and the lowering effect on HDL-C by ANGPTL3 inhibition is an EL-dependent mechanism.^17^ Additionally, EL can affect apoB-containing lipoprotein metabolism. EL overexpression was associated with a decrease in total and non-HDL-C levels in several mouse models, including *ldlr* KO mice.^18^ Interestingly, overexpression of EL was also associated with faster LDL particle clearance in *ldlr* KO mice,^18^ suggesting that the increase in LDL apoB catabolism observed during treatment with evinacumab in the 4 patients with hoFH could be mediated, at least in part, by an increase in EL activity. Indeed, 2 recent reports identified the critical role of EL in mediating LDL-C lowering by an LDLR independent pathway.^10,11^ These studies, in mice lacking both LDLR and EL, support the importance of the ANGPTL3/EL pathway in mediating VLDL remnant particle clearance and LDL-C lowering.^10^ The marked increase in IDL apoB FCR observed in our patients with hoFH after treatment with evinacumab are in line with those results. We also observed a small but consistent increase in LDL apoB production rate and a more substantial increase in LDL apoB FCR, suggesting that ANGPTL3 inhibition may also directly affect LDL apoB metabolism in patients with hoFH. Further research is needed to fully elucidate the mechanism of evinacumab-induced LDL-C lowering in humans.

Although the existence of an LDLR-independent pathway is supported by studies in mice^8,10^ as well as by the remarkable reduction in LDL-C observed in patients with hoFH treated with evinacumab^19,20^ and the increase in LDL apoB FCR in carriers of either *LDLR* null/null or defective variants observed in this study, it is not yet clear what receptor(s) are responsible for apoB-containing lipoproteins uptake from the circulation. Extensive studies in animal models suggest that the LDL-C lowering effect of evinacumab is not dependent on a number of other receptors or ligands, such as apolipoprotein E, LDLR-related protein 1, and syndecan 1, which are known to affect LDL or its precursors,^6^ and SR-BI.^10^

Alternative to an enhanced clearance, a reduction in LDL-C levels could theoretically be caused by a decreased production in LDL precursors (ie, VLDL and/or IDL). Although the mechanism(s) underlying a decrease in VLDL secretion are not immediately apparent, kinetic studies in fasting carriers of *ANGPTL3* LOF mutations showed a significant decrease in VLDL apoB production rate.^1^ A similar finding was observed in hepatocarcinoma cell lines treated with ANGPTL3 siRNA.^7^ A decrease in VLDL-TG secretion, but not in VLDL apoB secretion or VLDL clearance, was observed in mouse models treated with a monoclonal antibody^6^ or antisense oligonucleotides^8^ against ANGPTL3. In our study, we observed a reduction in VLDL apoB secretion in the 2 subjects carrying 2 *LDLR* null variants but not in the 2 subjects carrying *LDLR* defective variants. Thus, the effect on VLDL production may differ based on the mechanism by which ANGPTL3 is inhibited (ie, via intrahepatic RNA inhibition or a monoclonal antibody), the genetic background (*ANGPTL3* LOF variant versus *ANGPTL3* wildtype with pharmacological ANGPTL3 inhibition; *LDLR* null versus *LDLR* defective variants), and metabolic state (fasting versus nonfasting) of the studied population. Larger studies are needed to investigate these hypotheses.

The diverse clinical and genetic characteristics in the 4 subjects warrant further discussion. Although a genetic defect was identified in all patients, the subjects from UPENN carried *LDLR null/null* variants, resulting in total loss of LDLR function.^19^ The baseline LDL-C levels were therefore higher compared with the levels in 2 patients from the AUMC, who carried *LDLR* defective variants^19^ and had LDL-C levels that were lower with the concomitant medications. It is of particular interest that, contrary to other lipid-lowering drugs, such as PCSK9 inhibitors,^21,22^ the lipid lowering effect of ANGPTL3 inhibition by evinacumab seems to be independent of the presence of residual LDLR activity, supporting the data obtained in animal models,^6,10^ as well as in a pilot clinical trial.^9^ These findings are confirmed in the recently published ELIPSE trial (Efficacy and Safety of Evinacumab in Patients With Homozygous Familial Hypercholesterolemia), which showed a similar LDL-C lowering effect of Evinacumab in patients with hoFH with *null/null* and non-*null/null* variants.^20^

We acknowledge the several limitations of our study. First and foremost, the sample size of 4 patients with hoFH is small. Because of the interpatient variability in kinetic parameters, we would need a greater number of patients to conduct formal statistical testing on these parameters. For this reason, the study is only descriptive. Second, all 4 subjects carried *LDLR* variants in the *LDLR* gene, and kinetic results may differ in hoFH carrying variants in other FH-causing genes, namely *APOB*, *PCSK9*, and *LDLRAP1*. Furthermore, different infusion and lipoprotein isolation protocols were used at the 2 participating centers and may have contributed to some variability in the fit of the compartmental model and, ultimately, to some variability in the kinetic results. Last, while 3 subjects underwent the on-treatment kinetic study 6 weeks after the evinacumab infusion, one of the AUMC subjects (AUMC_1) underwent the on-treatment kinetic study 1 week after receiving the infusion. Based on the data collected during the parent clinical trials and other studies, LDL-C levels typically reach nadir after 4 weeks of receiving evinacumab infusion and remain generally stable at week 6.^9^ Therefore, it is possible that subject AUMC_1 was not yet in a steady state when he underwent the second kinetic study, which may have contributed to some of the observed heterogeneity in the results. An early (non steady state) effect of evinacumab could have resulted in the observed magnitude of effect on IDL-apoB FCR of 1300% in this particular subject (see Figure). These limitations, taken together, have likely contributed to some of the variability observed in the kinetic parameters in this study, and may affect the generalizability of the results.

In conclusion, ANGPTL3 inhibition with evinacumab markedly increases IDL and LDL apoB catabolic rates in this small study of 4 patients with hoFH, suggesting that evinacumab lowers LDL-C predominantly by increasing apoB-containing lipoprotein clearance from the circulation. Additional studies with a larger sample size are needed to confirm our findings as well as to identify the biological pathways involved in this process.

## Acknowledgments

We thank all 4 patients for their participation in this study. We also thank Dr Darko Stefanovski for helpful discussion about the kinetic data analysis. Additional information: Coauthor Daniel A. Gipe, MD, who was instrumental in the research and drafting of the article, died June 29, 2019.

## Sources of Funding

This study was supported by Regeneron Pharmaceuticals. The research unit at the University of Pennsylvania (Center for Human Phenomic Science) was supported by the National Center for Advancing Translational Sciences of the National Institutes of Health under award number UL1TR001878. The content is solely the responsibility of the authors and does not necessarily represent the official views of the National Institutes of Health.

## Disclosures

L.F. Reeskamp is co-founder of Lipid Tools. J.S. Millar is in part supported by grants HL148769 and HL145437 from the National Institutes of Health. D.J. Rader serves on Scientific Advisory Boards for Alnylam, Novartis, Pfizer, and Verve. D.A. Gipe was an employee of Regeneron Pharmaceuticals. G.K. Hovingh has served as consultant and speaker for biotech and pharmaceutical companies that develop molecules that influence lipoprotein metabolism, including Regeneron, Pfizer, MSD, Sanofi, and Amgen. Until April 2019, G.K. Hovingh served as PI for clinical trials conducted with Amgen, Sanofi, Eli Lilly, Novartis, Kowa, Genzyme, Cerenis, Pfizer, Dezima, and Astra Zeneca; and with current and past research grants from ZonMW (ViDi 016.156.445), EU, AMGEN, Sanofi, AstraZeneca, Aegerion, and Synageva. The Department of Vascular Medicine, Amsterdam UMC, receives honoraria and investigator fees for sponsor driven studies/lectures for companies with approved lipid-lowering therapies in The Netherlands. Since April 2019, G.K. Hovingh is partly employed by Novo Nordisk (0.7FTE) and Amsterdam UMC (0.3FTE). M. Cuchel is in part supported by grants HL148769 and HL145437 from the National Institutes of Health. She has received institutional research funding for conducting clinical trials from Akcea Therapeutics, Regeneron Pharmaceuticals, and REGENXBIO and received Advisory Board honorarium from Amryt Pharma.

## Supplementary Material


