# Supplementary material for: ANGPTL3 Inhibition With Evinacumab Results in Faster Clearance of IDL and LDL apoB in Patients With Homozygous Familial Hypercholesterolemia—Brief Report
Source: Arterioscler Thromb Vasc Biol. 2021 Mar 11;41(5):1753–9. doi: 10.1161/ATVBAHA.120.315204 (PMC8057526; doi:10.1161/ATVBAHA.120.315204)
Supplement: Supplementary file 1 [file atv-41-1753-s001.docx]

**Supplemental Materials**

**ANGPTL3 inhibition with evinacumab results in faster clearance of IDL and LDL apolipoprotein B in homozygous familial hypercholesterolemia patients**

*Laurens F. Reeskamp^1^, John S. Millar^2,3^, Liya Wu^3^, Hans Jansen^4^, Dewi van Harskamp^5^, Henk Schierbeek^5^, Daniel A. Gipe^6^, Daniel J. Rader^3^, Geesje M. Dallinga-Thie^4^, G. Kees Hovingh^1^, Marina Cuchel^3^*

1. Department of Vascular Medicine, Amsterdam UMC, location AMC, University of Amsterdam, Amsterdam, The Netherlands
2. Institute for Diabetes, Obesity, and Metabolism, Perelman School of Medicine, University of Pennsylvania, Philadelphia, Pennsylvania, United States of America
3. Division of Translational Medicine and Human Genetics, Department of Medicine, Perelman School of Medicine, University of Pennsylvania, Philadelphia, Pennsylvania, United States of America
4. Department of Experimental Vascular Medicine, Amsterdam UMC, location AMC, University of Amsterdam, Amsterdam, The Netherlands
5. Stable Isotope Research Laboratory, Endocrinology, Amsterdam UMC, location AMC, University of Amsterdam, Vrije Universiteit, Amsterdam, The Netherlands
6. Regeneron Pharmaceuticals, Inc., Tarrytown, NY, United States of America

| **Contents** | **Page** |
| --- | --- |
| **Supplementary Table I:** Complete list of apoB kinetic parameters before and after evinacumab | 2 |
| **Supplementary Figure I:** ApoB kinetic compartmental model | 3 |
| **Supplementary Figure II:** Individual apolipoprotein B100 production rates and fractional catabolic rates | 4 |
| **Supplementary Figure IIIA:** Model fit for AUMC_1 | 5 |
| **Supplementary Figure IIIB:** Model fit for AUMC_2 | 6 |
| **Supplementary Figure IIIC:** Model fit for UPENN_1 | 7 |
| **Supplementary Figure IIID:** Model fit for UPENN_2 | 8 |
| **Major Resources Table** | 9 |

**Supplementary Table I:** Complete list of apoB kinetic parameters before and after evinacumab

| **Subject** | **AUMC_1** | | **AUMC_2** | | **PENN_1** | | **PENN_2** | | **Mean Percent Change (±SD)** |
| --- | --- | --- | --- | --- | --- | --- | --- | --- | --- |
| **Time point** | **Baseline** | **Treatment** | **Baseline** | **Treatment** | **Baseline** | **Treatment** | **Baseline** | **Treatment** |  |
| **Body weight (kg)** | 92.7 | 90.2 | 66 | 68 | 79.9 | 81.4 | 70.2 | 68.5 | 0 (3) |
| **Plasma apoB** | 78.83 | 42.54 | 73.65 | 44.57 | 190.2 | 119.8 | 381.8 | 192.4 | -43 (6) |
| **VLDL apoB concentration (mg/dl)** | 5.61 | 5.71 | 1.57 | 1.24 | 0.7 | 0.23 | 4.5 | 0.97 | -41 (38) |
| **IDL apoB concentration (mg/dl)** | 11.8 | 1.03 | 10.61 | 1.97 | 0.62 | 0.21 | 4.76 | 0.66 | -81 (11) |
| **LDL apoB concentration (mg/dl)** | 61.42 | 35.81 | 61.46 | 41.36 | 188.9 | 119.4 | 372.5 | 190.8 | -40 (7) |
| **VLDL apoB PR (mg/kg/d)** | 1.99 (0.38) | 2.36 (0.55) | 6.54 (0.18) | 7.65 (0.32) | 13.24 (4.70) | 4.74 (1.64) | 45.85 (17.45) | 12.23 (4.66) | -25 (50) |
| **IDL apoB PR (mg/kg/d)** | 2.02 (0.18) | 2.46 (0.31) | 4.36 (0.06) | 5.42 (0.10) | 8.36 (0.45) | 6.94 (0.46) | 9.91 (0.03) | 12.25 (0.10) | 13 (20) |
| **LDL apoB PR (mg/kg/d)** | 6.35 (0.65) | 8.29 (1.11) | 12.17 (0.36) | 15.93 (0.68) | 20.34 (2.47) | 26.9 (4.80) | 26.31 (1.88) | 30.37 (3.75) | 27 (8) |
| **VLDL apoB FCR (pools/d)** | 0.79 (0.15) | 0.92 (0.21) | 9.24 (0.26) | 13.74 (0.57) | 45.12 (16.03) | 44.01 (15.25) | 14.08 (5.36) | 14.1 (5.37) | 16 (24) |
| **IDL apoB FCR (pools/d)** | 0.38 (0.03) | 5.32 (0.67) | 0.91 (0.01) | 6.12 (0.12) | 30.9 (1.66) | 57.92 (3.82) | 4.15 (0.01) | 25.12 (0.20) | 616 (504) |
| **LDL apoB FCR (pools/d)** | 0.23 (0.02) | 0.51 (0.07) | 0.44 (0.01) | 0.86 (0.04) | 0.24 (0.03) | 0.50 (0.09) | 0.16 (0.01) | 0.36 (0.04) | 113 (14) |

apoB, apolipoprotein B100; VLDL, very low-density lipoprotein; IDL, intermediate-density lipoprotein; LDL, low-density lipoprotein; PR, production rate; FCR, fractional catabolic rate. PR and FCR are shown as parameter estimates (standard deviation).

**Supplementary Figure I:** Multicompartmental model used to analyze tracer kinetic data. The model is the same as that used in previous studies in patients with homozygous FH^1^ with the addition of a parameter for direct transfer of apoB from small VLDL to LDL. To improve parameter identifiability, the parameters representing clearance from the VLDL and IDL remnant pools and that representing clearance of LDL apoB were determined using Bayesian estimation.


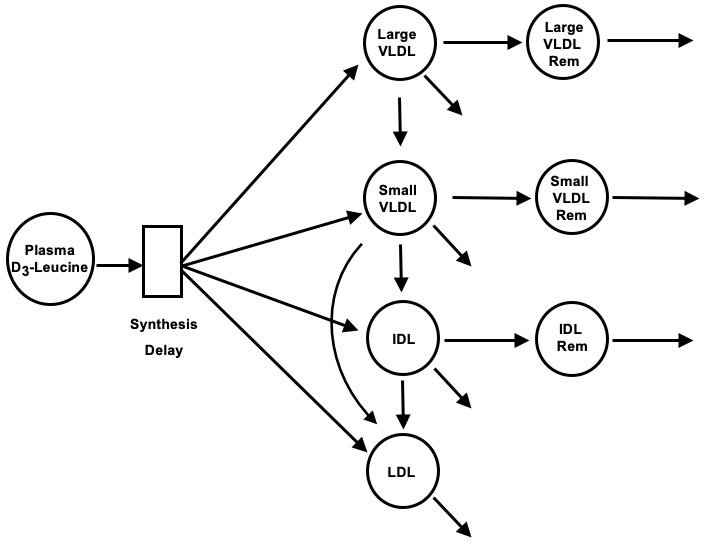


**Supplementary Figure II:** Individual apolipoprotein B100 production rates and fractional catabolic rates. Values of apolipoprotein B100 (apoB) production rates (PR) and fractional catabolic rates (FCR) for individual subjects before and after treatment with evinacumab. VLDL, very-low density lipoprotein; IDL, intermediate-density lipoprotein; LDL, low-density lipoprotein.


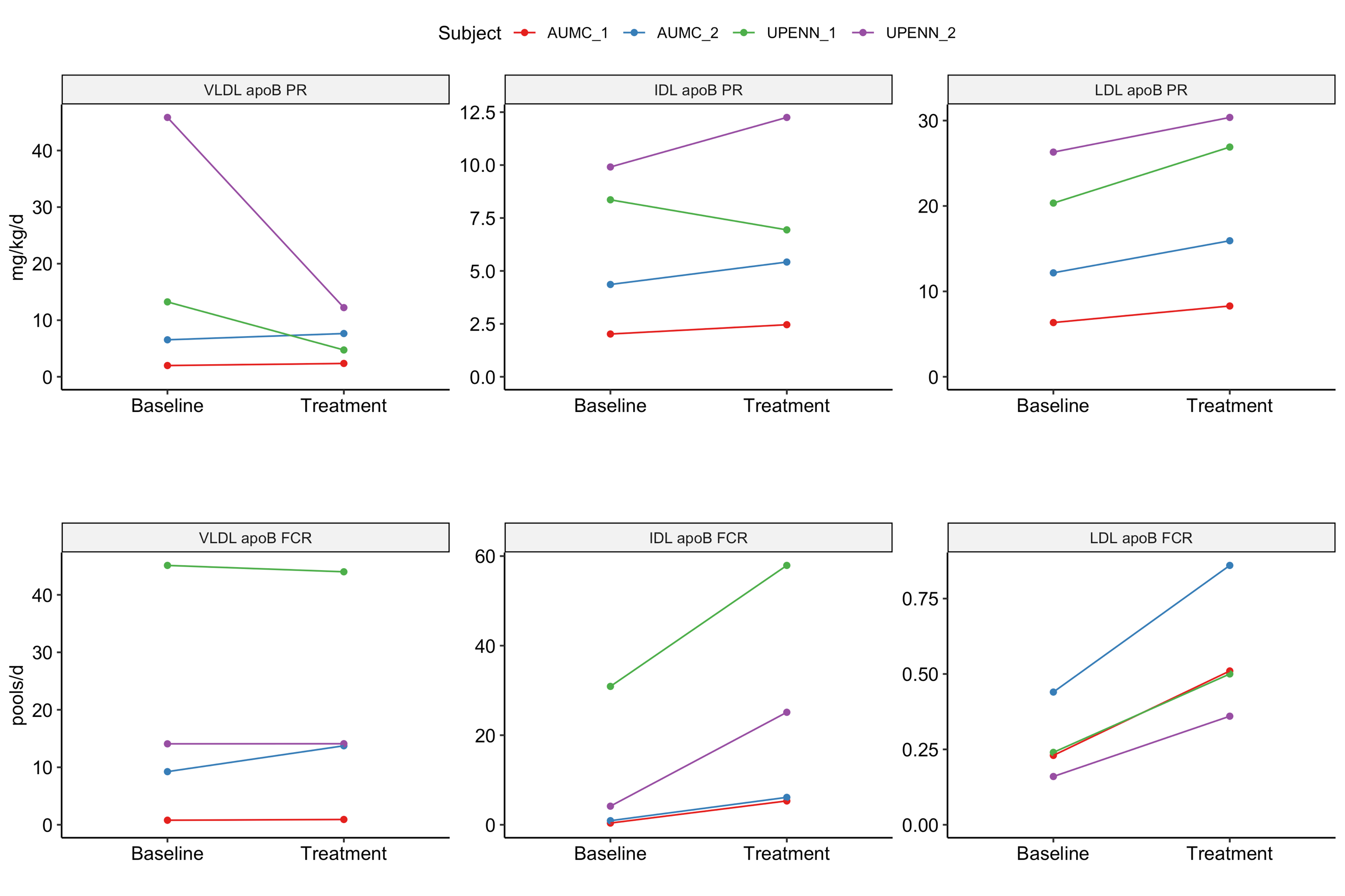


**Supplementary Figure IIIA:** Model fit (line) to tracer data (triangles) for AUMC_1


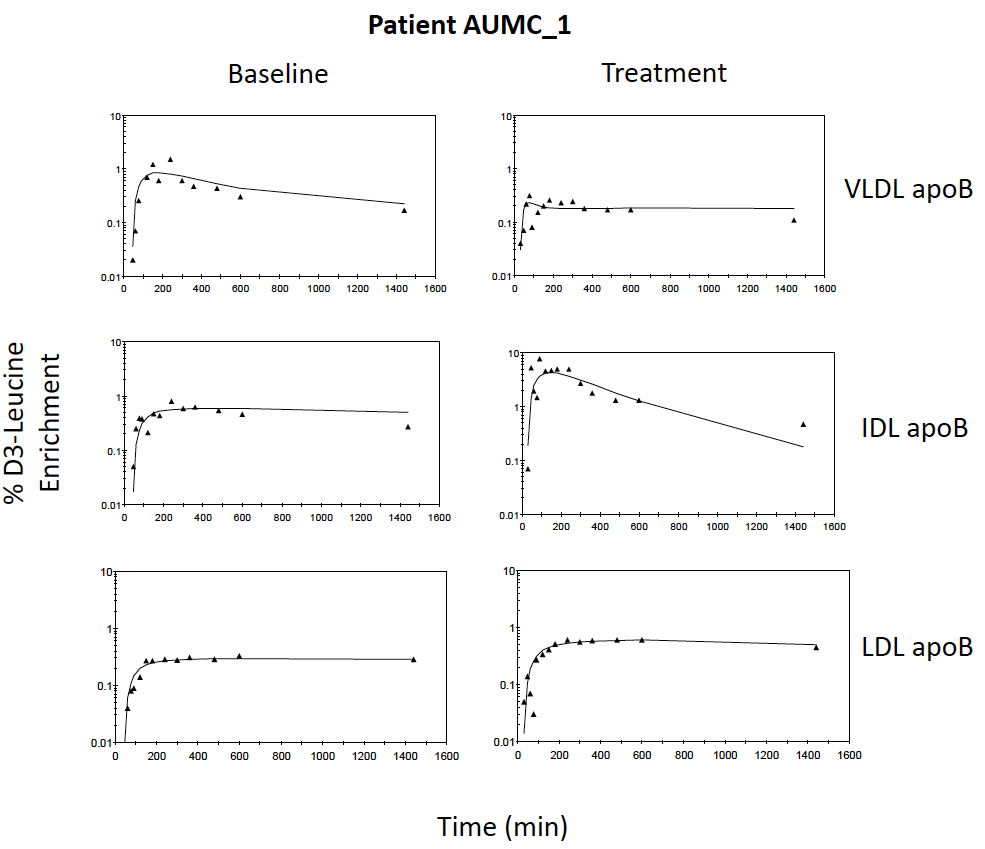


**Supplementary Figure IIIB:** Model fit (line) to tracer data (triangles) for AUMC_2


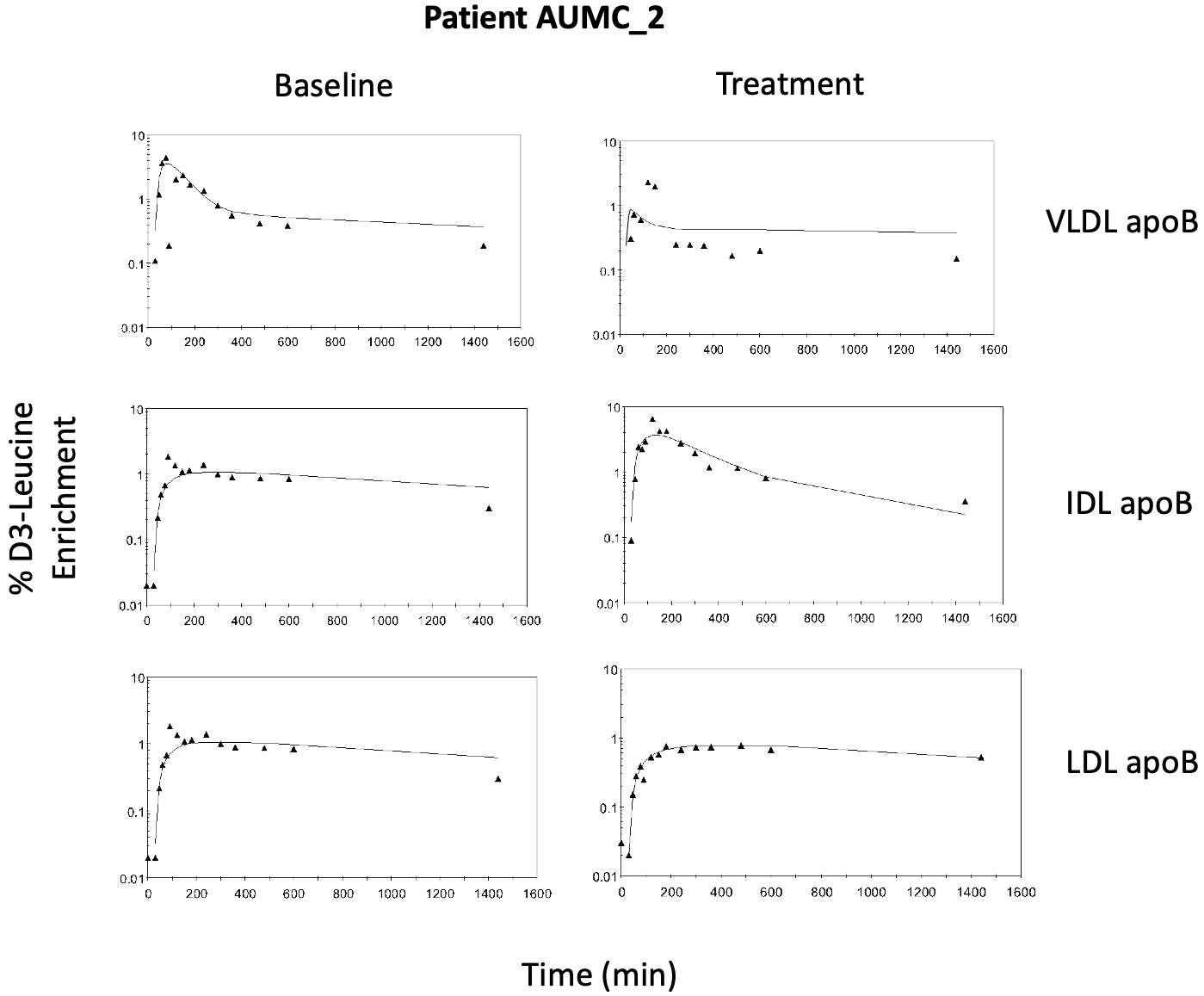


**Supplementary Figure IIIC:** Model fit (line) to tracer data (triangles) for UPENN_1


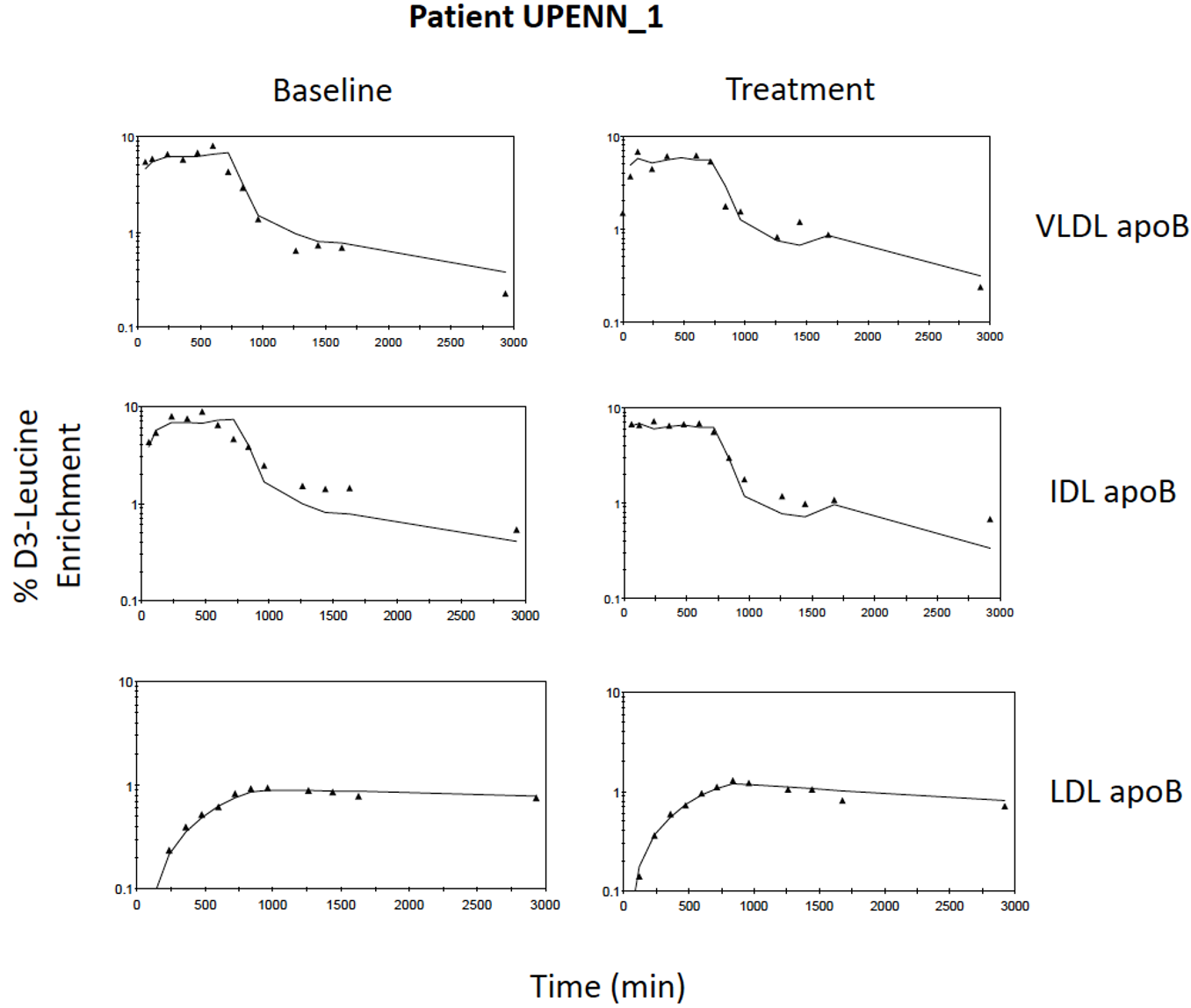


**Supplementary Figure IIID:** Model fit (line) to tracer data (triangles) for UPENN_2


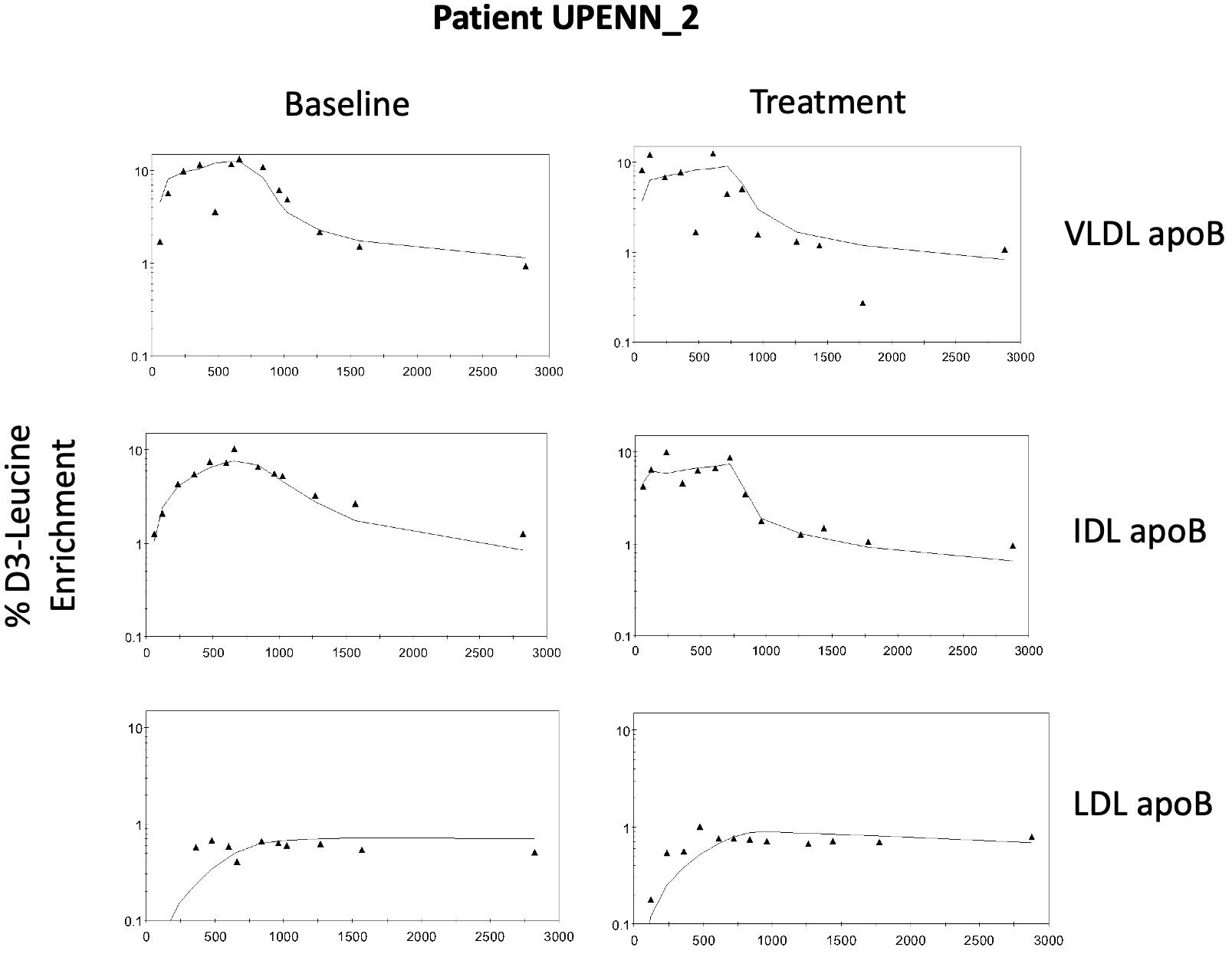


**Major Resources Table**

In order to allow validation and replication of experiments, all essential research materials listed in the Methods should be included in the Major Resources Table below. Authors are encouraged to use public repositories for protocols, data, code, and other materials and provide persistent identifiers and/or links to repositories when available. Authors may add or delete rows as needed.

**Data & Code Availability**

| **Description** | **Source / Repository** | **Persistent ID / URL** |
| --- | --- | --- |
| R-code for summary data, tables, and figures | First author |  |
| Kinetic modelling code | Corresponding author |  |
| Raw data | Corresponding author |  |

**Other**

| **Description** | **Source / Repository** | **Persistent ID / URL** |
| --- | --- | --- |
| (5,5,5-^2^H_3_)-leucine | Cambridge Isotope Laboratories | #DLM-1259-MPT |
| Evinacumab (fully human monoclonal antibody against ANGPTL3) | Regeneron Pharmaceuticals |  |
