# Supplementary figures and images for: ANGPTL3 Inhibition With Evinacumab Results in Faster Clearance of IDL and LDL apoB in Patients With Homozygous Familial Hypercholesterolemia—Brief Report
Source: Arterioscler Thromb Vasc Biol. 2021 Mar 11;41(5):1753–9. doi: 10.1161/ATVBAHA.120.315204 (PMC8057526; doi:10.1161/ATVBAHA.120.315204)

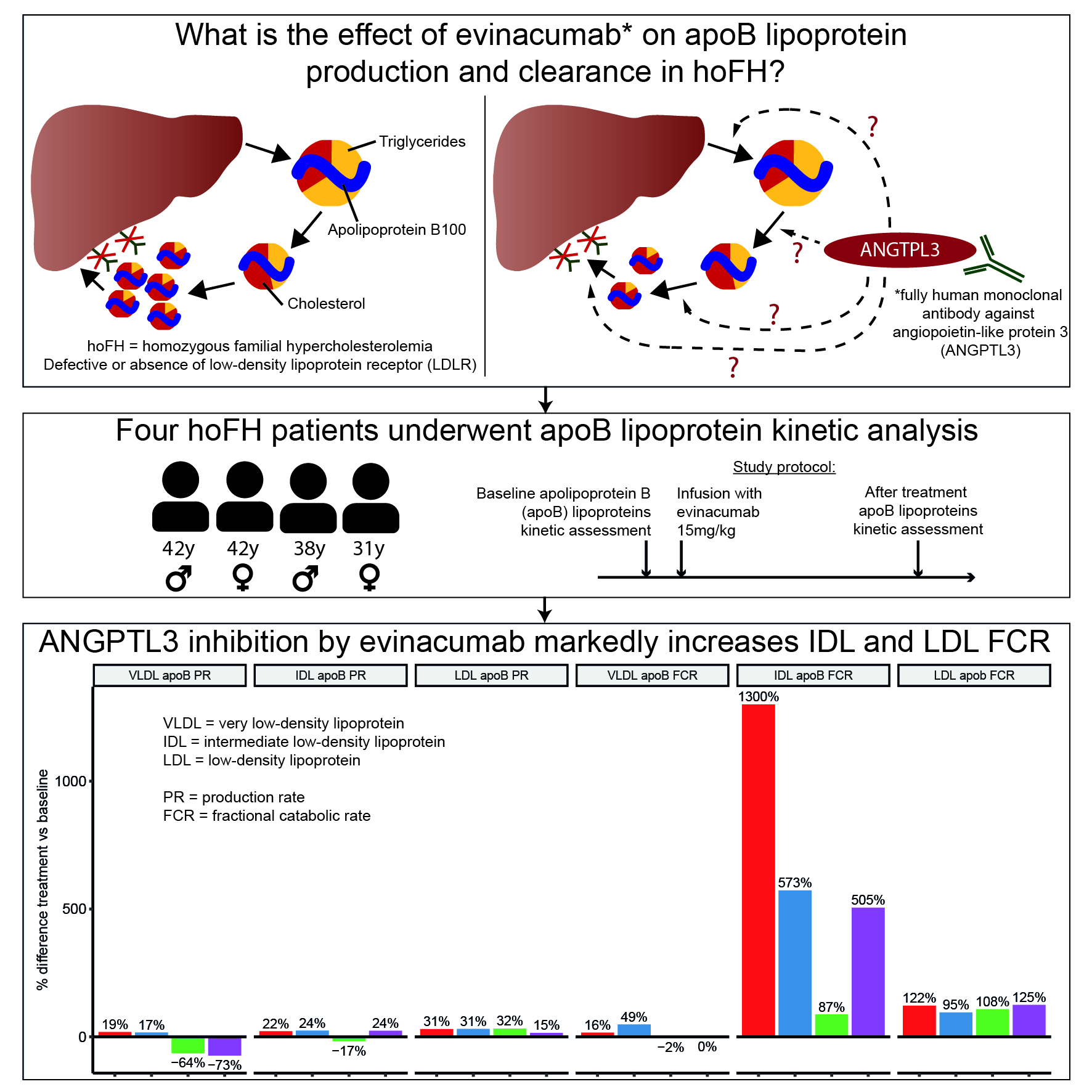

Supplement: Supplementary file 2 [file atv-41-1753-s002.jpg]
